# In-source freezing of *Trifolium repens* leaf tissue to increase spatial resolution and quench stress responses during IR-MALDESI mass spectrometry imaging

**DOI:** 10.1007/s00216-026-06710-z

**Published:** 2026-08-03

**Authors:** Quinn Mills, David C. Muddiman

**Affiliations:** Biomolecular Imaging Laboratory for Disease and Exposure Research (BILDER), Department of Chemistry, North Carolina State University, Raleigh, NC USA

**Keywords:** IR-MALDESI, Mass spectrometry imaging, Plant imaging, Plant metabolomics, Plant stress response

## Abstract

Mass spectrometry imaging (MSI) is a robust analytical tool for resolving the spatial localization of diverse metabolites in a tissue sample simultaneously. Infrared matrix-assisted laser desorption electrospray ionization (IR-MALDESI) is particularly advantageous for analyzing leaf tissue, since it bypasses the cuticle and uses endogenous water in the sample as a matrix, avoiding delocalization and reducing costs associated with sample preparation. While direct analysis of fresh tissue offers substantial benefits, it also presents a less-discussed challenge: plants initiate a cascade of metabolic responses to various forms of stress (e.g., mechanical wounding, heat stress), so freshly-harvested tissue can undergo major metabolic changes before and during analysis. Herein, a method is introduced which retains the benefits of IR-MALDESI MSI but increases spatial resolution and quenches metabolic processes by quickly freezing leaf tissue within the source enclosure immediately after harvest. Leaf samples were mounted on a glass slide with carboxymethylcellulose gel immediately after harvest and moved into the source enclosure, humidity was purged, and the sample was frozen using the Peltier-cooled stage. Camera images demonstrated minimal ice deposition on the sample surface, and evaluation of the phenylpropanoid pathway suggested that freezing the sample inhibited post-harvest stress responses during and prior to analysis. Frozen tissue analysis significantly decreased ablation spot diameter from 65.4 to 43.9 µm, enabling approximately a 1.5-fold increase in spatial resolution with a conventional 2.97-µm IR laser.

**Graphical Abstract:**

**Figure:**
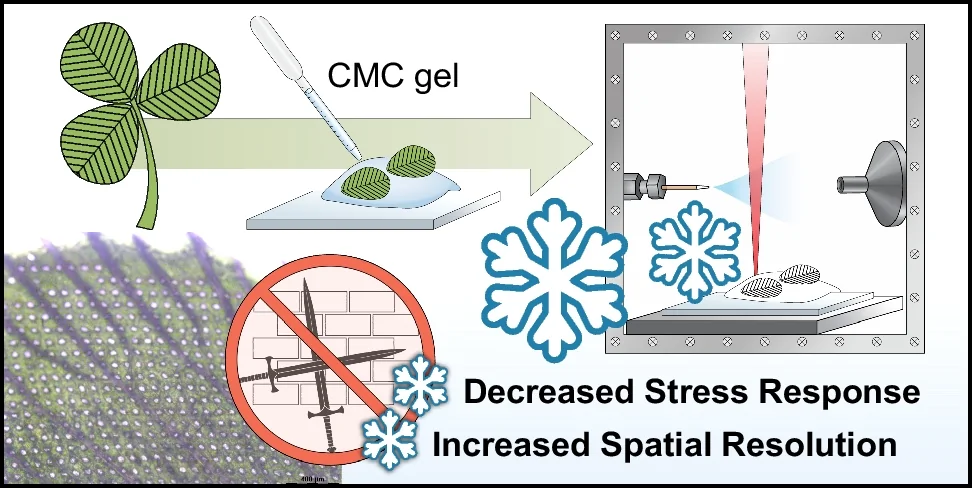

**Supplementary Information:**

The online version contains supplementary material available at https://doi.org/10.1007/s00216-026-06710-z.

## Introduction

Mass spectrometry (MS) is a common and crucial tool for identifying molecular species in metabolomics analyses, particularly in untargeted studies on plants, which can contain hundreds of metabolites. These studies offer important insight into secondary metabolites responsible for stress signaling, host–pathogen relationships, growth and development, and other processes with major implications for agriculture, plant science, conservation, and medicine. Due to the expansive variety of metabolites present, MS-based plant metabolomics typically focuses on LC–MS and GC–MS hyphenated techniques, discarding spatial information in favor of more robust untargeted analysis from separations [1]. While coupling MS to chromatographic techniques is often necessary to obtain informative MS/MS data from complex samples, spatial information obtained through mass spectrometry imaging (MSI) is invaluable for deepening our understanding of the complex metabolic relationships between analytes. In MSI analysis, mass spectra are collected from an array of voxels across a sample, and the ion abundance of a chosen *m*/*z* can be extracted for each pixel to form a heat map. Hundreds of metabolites can be spatially resolved in a single MSI experiment, and colocalized features can indicate biosynthetic precursors or other metabolic interactions [2–5], gradients of metabolites (e.g., auxins) can reveal growth patterns [6–8], and accumulation in different tissues can elucidate storage mechanisms [9, 10 ]. MSI methods have been thoroughly developed for analysis of mammalian tissues [11]; however, there is significant room for growth and standardization in spatial metabolomics of plants, partially due to complications from their structure and chemical complexity [12].

Compared to mammalian samples, plants have an increased water content, highly heterogeneous tissues, a brittle structure due to cell walls, and a hydrophobic cuticle covering leaf surfaces [13]. Many novel solutions have been introduced to address the resulting MSI challenges: embedding and cryosectioning protocols have been optimized for diverse plant tissues [14, 15 ], certain metabolites have been imprinted onto membranes or other surfaces for indirect analysis [10, 16,17 ], and automatic *z*-axis corrections have been applied to image non-flat samples [18–20]. Different ionization methods can also be leveraged for distinct applications, such as surface analysis of leaves by ultraviolet matrix-assisted laser desorption ionization (UV-MALDI) or desorption electrospray ionization (DESI), which takes advantage of their lack of penetration through the cuticle [21, 22 ]. Alternatively, the infrared lasers in infrared atmospheric pressure MALDI (IR-AP-MALDI) [23] and infrared matrix-assisted laser desorption electrospray ionization (IR-MALDESI) [24] ablate through the cuticle to access deeper tissue layers for a more comprehensive analysis.

A unique advantage of IR-MALDESI analysis is the use of endogenous water in the sample as a matrix, enabling direct analysis of fresh leaf tissue without any sample preparation. Complex sample preparation methods can compromise sample integrity and delocalize analytes of interest [25–27], and even basic freeze–thaw cycles are known to cause delocalization, particularly of hydrophilic metabolites [14]. Delocalization from freeze–thaw cycles is especially pronounced in leaves: vacuoles in the cells store large amounts of water, causing the cells to rupture upon freezing. When the sample thaws, cell contents that were expelled beyond the cuticle during freezing are further delocalized by its hydrophobic nature. MALDI methods commonly lyophilize plant samples immediately after flash-freezing to minimize delocalization by removing water before thawing [28], but lyophilization removes important (semi)-volatile species [29]. While IR-MALDESI conveniently bypasses these challenges by analyzing fresh tissue, it does not address a crucial outcome of flash-freezing: preventing metabolic changes resulting from stress during sample harvest, storage, and analysis.

Stress responses in plants are known to cause rapid metabolic changes and are becoming a major area of metabolomics research [30]. In response to mechanical wounding, herbivory, heat, and water loss, plants can activate diverse signaling pathways (e.g., jasmonate, salicylic acid, and ethylene) and biosynthetic pathways (e.g., the phenylpropanoid pathway) to defend against stress [30–32]. In MSI analyses of mammalian tissues, samples are often flash-frozen, freeze-dried, and/or treated with protease inhibitors immediately after harvest to prevent native enzymatic activity or protein degradation [33, 34 ]. When extractions are prepared from plant tissues for GC–MS or LC–MS analyses, samples are also conventionally flash-frozen after harvest [35]. To balance the benefits of IR-MALDESI with the need to quench stress responses for biologically accurate metabolomics data, a method was developed herein for freezing leaf samples in the MALDESI source immediately after harvest and maintaining frozen conditions throughout MSI analysis. In-source freezing without thawing minimizes delocalization from freeze–thaw cycles while also preventing unwanted stress responses.

Incidentally, this method also addresses a more thoroughly explored constraint of IR-MALDESI MSI. Combined with the inherent physical limits of an IR-wavelength laser, the high water content in plant tissue results in wide ablation spots, reducing spatial resolution. Several adjustments have already enabled higher spatial resolution for MALDESI MSI of leaves: the optical train was optimized with a reflective objective [36], AzC was incorporated to reduce spot-to-spot variability [19], and UV-MALDESI has been explored as an alternative method [37, 38 ]. While UV-MALDESI MSI has achieved the highest resolution to date and is a highly promising new route, it is not universally applicable, since it relies on an even distribution of endogenous flavonoids as a matrix and does not fully ablate through tissue. For experiments outside the scope of UV-MALDESI MSI, this study also presents in-source freezing of leaf tissue as a method to increase spatial resolution with conventional IR-MALDESI MSI.

## Methods

### Materials

LC–MS grade solvents and reagents (acetonitrile [ACN], formic acid [FA], and water) and plain glass microscope slides (1 mm) were purchased from Fisher Scientific (Nazareth, PA, USA). Carboxymethylcellulose (CMC) sodium salt was purchased from Sigma-Aldrich (St. Louis, MO, USA). Masking tape was purchased from Amazon (Seattle, WA, USA). Burn paper was purchased from ZAP-IT (Barrington, NJ, USA). All elements of the optical train (− 75 mm plano-concave beam expander [LC5401-E], + 250 mm plano-convex lens [LA5255], and Schwarzschild-like reflective objective [LMM15X-P01]) were purchased from Thorlabs (Newton, NJ, USA). Nitrogen gas was purchased from Arc Gases (Raleigh, NC, USA). All elements of the chromatic confocal probe (CA probe) system were purchased from Precitec GmbH & Co. KG (Gaggenau, Germany). Microscopes were purchased from Fisher Scientific (2 × images; 12–562-16, Waltham, MA, USA), and Leica Microsystems (5 × and 10 × images; LMD 7000, Wetzlar, Germany).

### Reagent preparation

The electrospray solvent was prepared as 50% (v/v) ACN attenuated with 0.2% (v/v) FA. This composition reflects literature precedent for IR-MALDESI analysis of leaves [19, 36 ]. The 5% (w/v) CMC gel was prepared with CMC sodium salt and LC–MS grade water. Concentrations of 2.5% and 5% CMC were tested based on established embedding protocols [13, 14 ] (liquid gelatin is not conducive to fast freezing), and the 5% CMC gel was selected for its ability to maintain structure while mounting samples.

### Leaf samples

White clover (*Trifolium repens*) was selected as a model due to (1) its accessibility in the region, (2) biological similarity between leaflets, (3) leaf thickness, and (4) metabolome coverage in the KNApSAcK database [39]. Samples were available within a 1-min walk of the lab, minimizing stress responses between harvest and the onset of MSI analysis. Preliminary data on frozen collards (Fig. S1) demonstrated incomplete ablation, so this experiment sought results on thinner leaves; the lamina of the clover leaves was approximately 0.10–0.15 mm. Biological similarity between leaflets allowed inference of metabolomic changes due to stress responses between analysis of leaflets 1 and 2 for each sample, and the 91 metabolites previously documented in *T. repens* in the KNApSAcK database allowed tentative identification of metabolites for data analysis.

Leaves were collected from the North Carolina State University campus after identification as *T. repens* based on morphological features including its prostrate growth, green trifoliolate leaves with light V-shaped marks, and globular racemes of 20–40 white florets [40].

### Frozen tissue analysis method

**Fig. 1:**
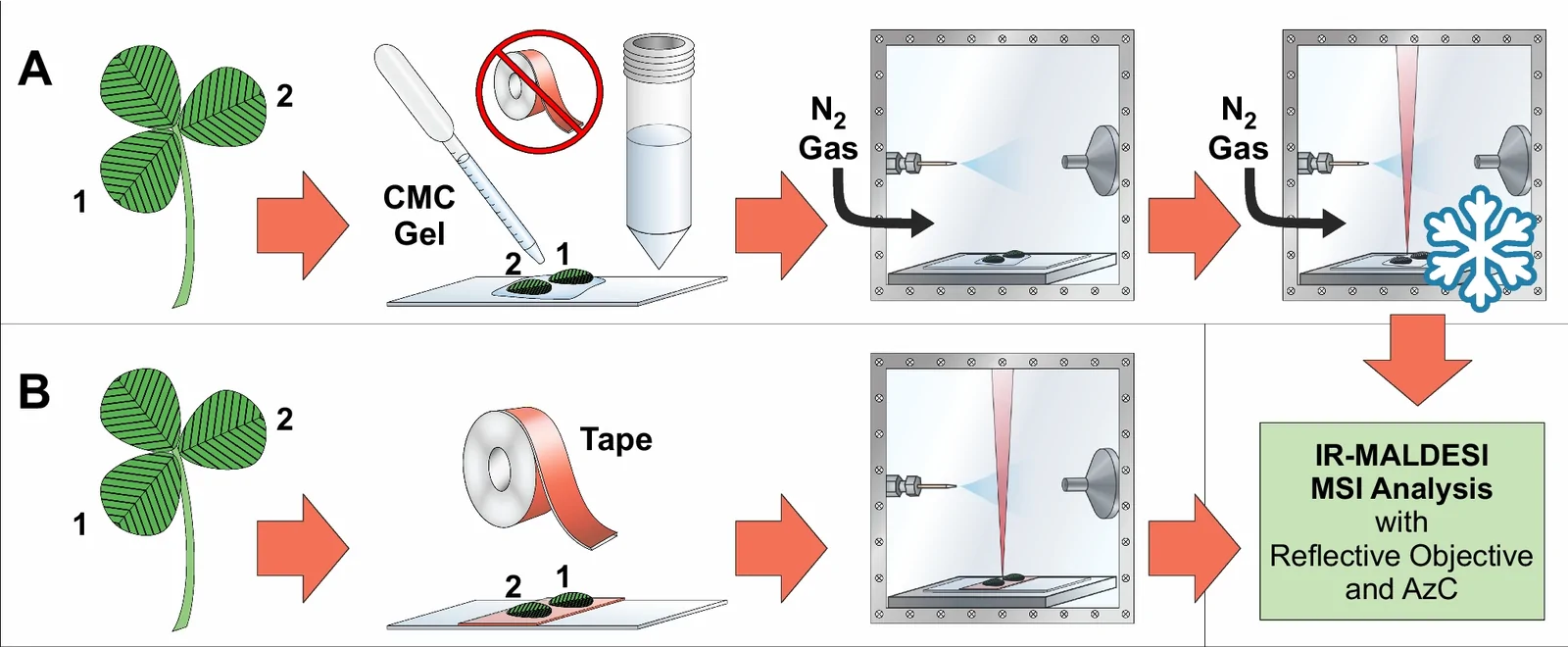
Experimental workflow for IR-MALDESI MSI analysis of clover leaves by **A** frozen analysis on CMC or **B** fresh analysis on tape (conventional method). For either method, two opposite leaflets were harvested from a clover leaf and mounted on a glass slide. One ROI was analyzed for each leaflet, and the two ROIs from each sample were analyzed consecutively

For frozen analysis, the source enclosure was first purged with nitrogen gas to 1–2% humidity. Immediately after leaves were harvested, leaflets were mounted on 5% CMC gel on a glass slide at room temperature, and the slide was placed on the Peltier-cooled stage. The enclosure was then purged back to 1–2% humidity, and the stage was cooled to − 10 °C to freeze the sample. Sample preparation required roughly 5 min from harvest to freezing. Once the gel froze, the leaflet appeared to freeze near-instantaneously—the gel was considered frozen once it transitioned from transparent to opaque, and the leaflet was considered frozen once the primary vein transitioned from translucent to opaque. These conditions were maintained throughout analysis. This process is summarized in Fig. 1A, as compared to the conventional workflow summarized in Fig. 1B.

### Experimental design

Clover leaves were harvested immediately prior to analysis; the total time between harvest and initiation of the RastirZ analysis sequence was approximately 5 min for fresh analysis, and the total time between harvest and initiation of freezing was approximately 5 min for frozen analysis. Fresh analysis was attempted on CMC as a control to investigate ion suppression, but the CMC dried during analysis, invalidating *z*-axis corrections and subsequently inhibiting ablation. These issues are summarized with Fig. S2 in Supplemental Material. Although the full sample could not be analyzed, ablated regions were used to rule out ion suppression from CMC. Fresh tissue analysis was conducted according to the current protocol for leaf analysis by IR-MALDESI-MSI [19]. Forceps were cleaned with methanol and used to mount samples on tape attached to a glass slide.

### IR-MALDESI MSI analysis

Samples were analyzed on the NextGen IR-MALDESI source, wherein the emitter tip is positioned coaxial to the mass spectrometer inlet and orthogonal to the sample stage. The electrospray solvent was flowed through the emitter tip at 1.5 µL/min, and a high voltage (3600 V) was applied to form an electrospray plume. All analyses occurred at atmospheric pressure and ambient temperature, and fresh tissue analysis was performed at ambient humidity. A 2.97-µm laser from JGM Associates, Inc. (Burlington, MA, USA) was fitted to a previously optimized optical train featuring a Schwarzschild-like reflective objective [36, 41 ]. The laser was tuned to deliver ~ 0.45 mJ/burst. Automatic z-axis corrections (AzC) accounted for sample topography using the CA probe and AzC method established by Xi et al. [19, 42 ].

The source was coupled to an Orbitrap Exploris 240 mass spectrometer (Thermo Fisher Scientific, Bremen, Germany). Mass spectra were collected in positive ionization mode over a range of *m*/*z* 100–1000, with the following instrument settings: small molecule mode, centroid data, 70% S-lens RF-level, 240,000_FWHM_ resolving power at *m*/*z* 200, and 15 ms ion injection time. EASY-IC was enabled, and automatic gain control (AGC) was disabled. Prior to MSI analysis, routine mass calibration was performed using Flexmix (Pierce Biotechnology, Rockford, IL).

### Data analysis

The diameters of randomly sampled ablation spots were measured using the “measure” feature of the Leica microscope. These measurements were compiled in Excel, and violin plots with overlaid boxplots were generated in RStudio. MSI datafiles were converted from Thermo Fisher .RAW to .mzML using MSConvert [43], and from .mzML to .imzML using imzMLConverter [44]. A list of known metabolites from *T. repens* was exported from the KNApSAcK database [39], and a list of corresponding *m*/*z* values was generated in Excel for [M + H]^+^, [M + Na]^+^, and [M + K]^+^ adducts. The .imzML files were uploaded to MSiReader v3.13 for analysis [45], which was used to generate ion images, abundances, and volcano plots for the list of *T. repens m*/*z* values. Bar graphs of detected vs. non-detected metabolites were generated in Excel.

## Results and discussion

**Fig. 2:**
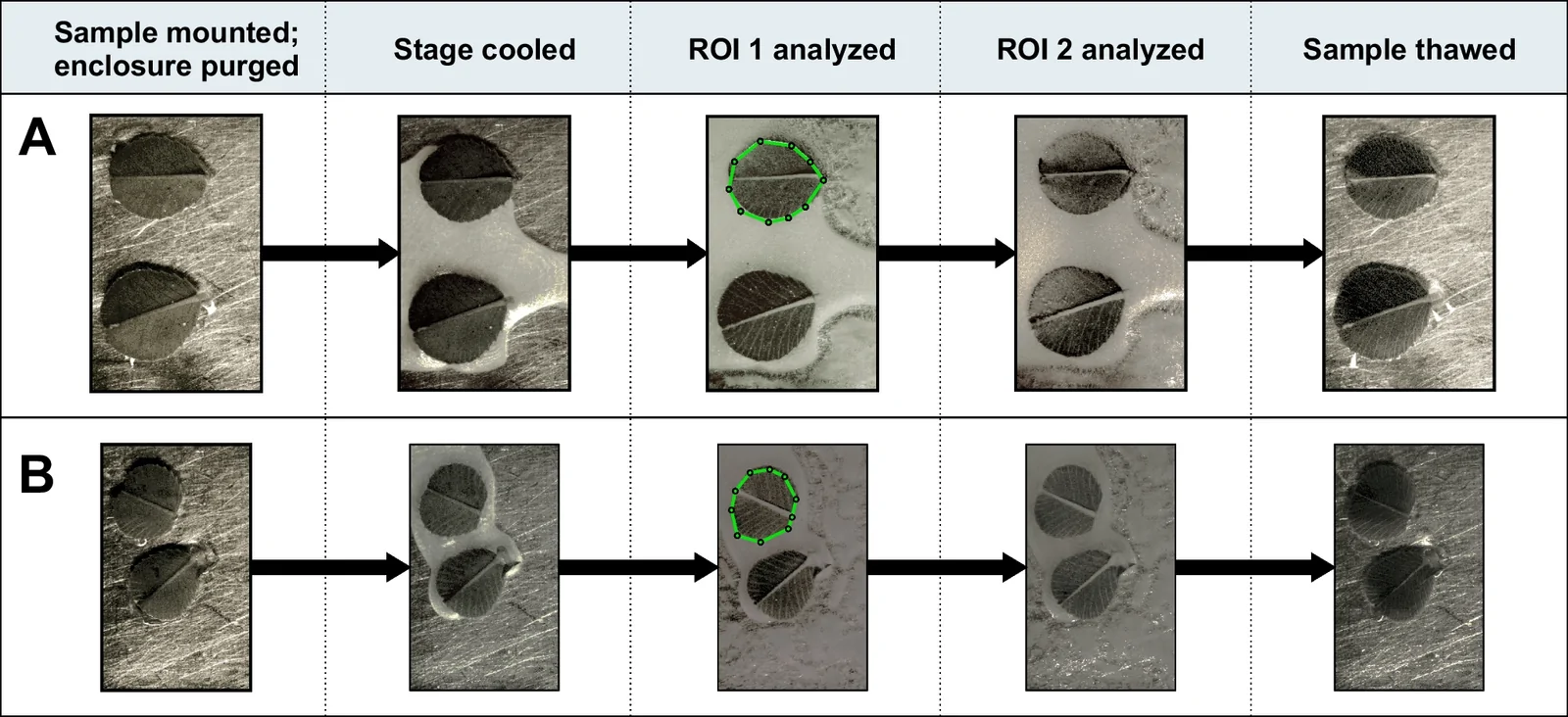
Camera images throughout IR-MALDESI analysis of frozen tissue for **A** the first pair of frozen clover leaflets, and **B** the second pair of frozen clover leaflets. From left to right, images were taken of the samples after the enclosure was purged and they were mounted on the CMC, after they were frozen via the Peltier-cooled stage, after ROI 1 (first leaflet, outlined in green) was analyzed, after ROI 2 (second leaflet) was analyzed, and once the sample was thawed after analysis

Although the leaf cuticle is notably ablated by IR-MALDESI, its hydrophobic nature prevents deposition of a consistent exogenous ice layer, which is often incorporated for cryosectioned mammalian tissues. Since it cannot be evenly distributed across the leaf surface, ice formation should be prevented to avoid matrix effects. Figure 2 includes camera images across critical points of the freezing and analysis process for the first frozen leaf sample (Fig. 2A) and second frozen leaf sample (Fig. 2B). The CMC gel was clear in the initial images after the samples were mounted and the enclosure was purged, but it became opaque in the next set of images, indicating that it froze when the stage was cooled. The primary veins of the leaflets also became more opaque, indicating that the leaf tissue froze successfully. Prior to analysis of the region-of-interest (ROI) on the first leaflet (ROI 1), there was no visual evidence of ice deposition on the leaves, but after analysis of the first leaflet in each pair, scattered ice crystals became visible only on the first leaflet. Similarly, ice crystals only became visible on the second leaflet (ROI 2) after it was analyzed. Ablation through the cuticle likely allowed remaining water vapor to crystallize onto newly-hydrophilic areas where the cuticle had been ablated. Ice was not visible on the second leaflet of each pair until after both leaflets were analyzed, so matrix effects during MSI analysis should have been minimal.

Although scattered ice crystals may form over the course of analysis and result in matrix effects, leaves also continuously lose water over the course of IR-MALDESI analysis of fresh tissue—water can be lost through severed vascular tissue and can freely evaporate from surfaces after the laser penetrates the cuticle. Since ablation relies on endogenous water, water loss would similarly result in matrix effects. Additionally, the resulting tissue shrinkage can pull the sample surface out of focus of the laser since AzC are determined for the whole sample prior to analysis; thus, water loss during fresh tissue analysis can also result in inconsistent ablation. Considering that both methods are predicted to result in minor matrix effects, frozen tissue analysis may be preferable since (1) it preserves the original distribution of water as the endogenous matrix, (2) it maintains effective AzC, and (3) randomly scattered ice crystals should result in less systematic bias than consistent water loss over time.

**Fig. 3:**
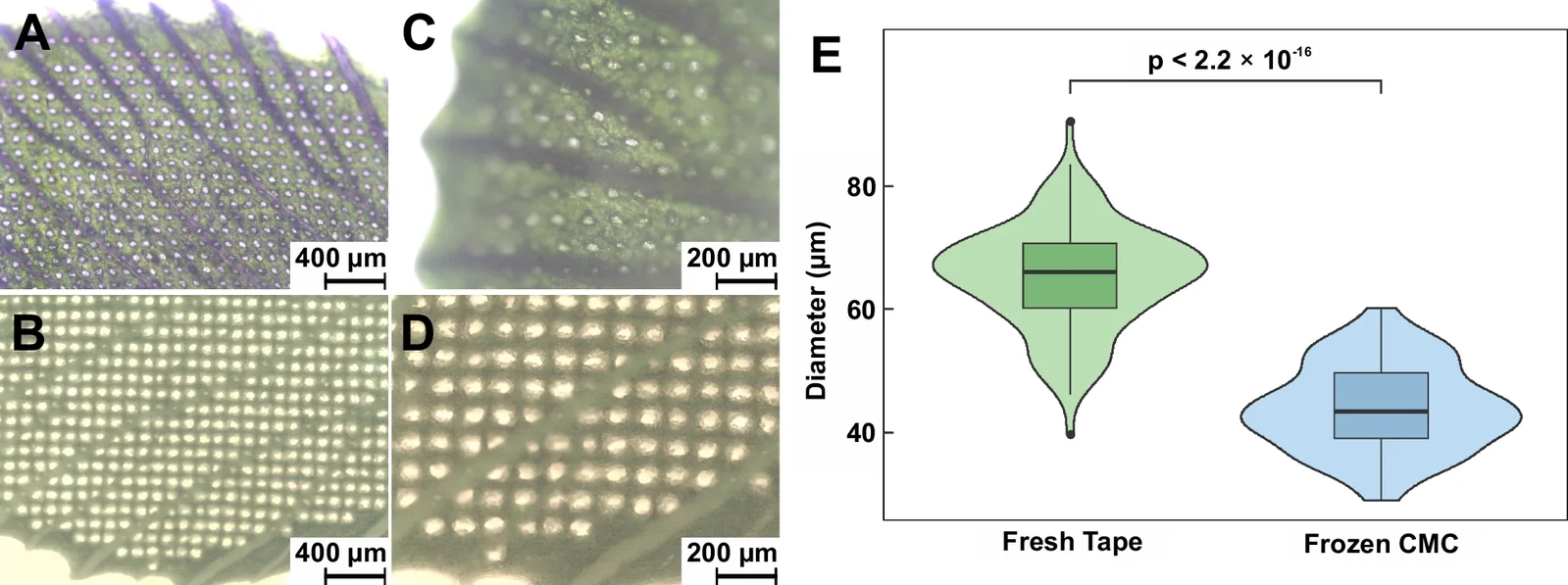
Images compare ablation spots at 5X magnification for **A** frozen analysis and **B** fresh analysis, and 10 × magnification for **C** frozen and **D** fresh analysis. **E** Differences in ablation spot diameter in violin plots and overlaid boxplots for the fresh (left) and frozen (right) methods. The Welch’s paired *t*-test gave *p* < 2.2 × 10^−16^ at the 95% confidence level

To enable increased spatial resolution while maintaining undersampling, it is crucial to reduce the diameter of ablation spots. In conventional IR-MALDESI analysis of fresh leaf tissue, high water content and tissue heterogeneity result in inconsistent ablation and large ablation spots, reducing both biological accuracy and spatial resolution. Applying a reflective objective can significantly reduce ablation spot diameter [36], and AzC can improve the overall consistency of ablation [19], but there remains room for improvement. In IR-MALDESI MSI of mammalian tissue, the reflective objective originally reduced ablation spot diameter from ~ 150 to ~ 55 µm [41] and then to 20 µm with a novel 2.94-µm laser [46]; however, the average ablation spot diameter of fresh leaf tissue has only reached ~ 146 µm for IR-MALDESI [36] and ~ 32 µm for UV-MALDESI [37]. In this study, the tissues were intentionally undersampled to ensure that the ablation spot diameters could be accurately measured under the microscope after analysis. In Fig. 3A, the ablation spots from the frozen analysis were visibly smaller than those in Fig. 3B from fresh analysis. Additionally, higher magnification revealed less thermal damage around the edges of the ablation craters in Fig. 3C for frozen analysis compared to Fig. 3D for fresh analysis. As shown in Fig. 3E, the frozen tissue analysis method significantly reduced the ablation spot diameter (*p* < 2.2 × 10^−16^ at 95% CI). When analyzing fresh tissue mounted on tape, the average diameter was 65.4 ± 19.5 µm, but the average diameter for frozen tissue on CMC was 43.9 ± 23.3 µm. Interestingly, this finding differs from the original study on mammalian tissue by Joignant et al., which found no significant difference between spot diameters on thawed and frozen tissue with the reflective objective [41]. This discrepancy is attributed to the differences in tissue composition between leaf and mammalian samples.

**Fig. 4:**
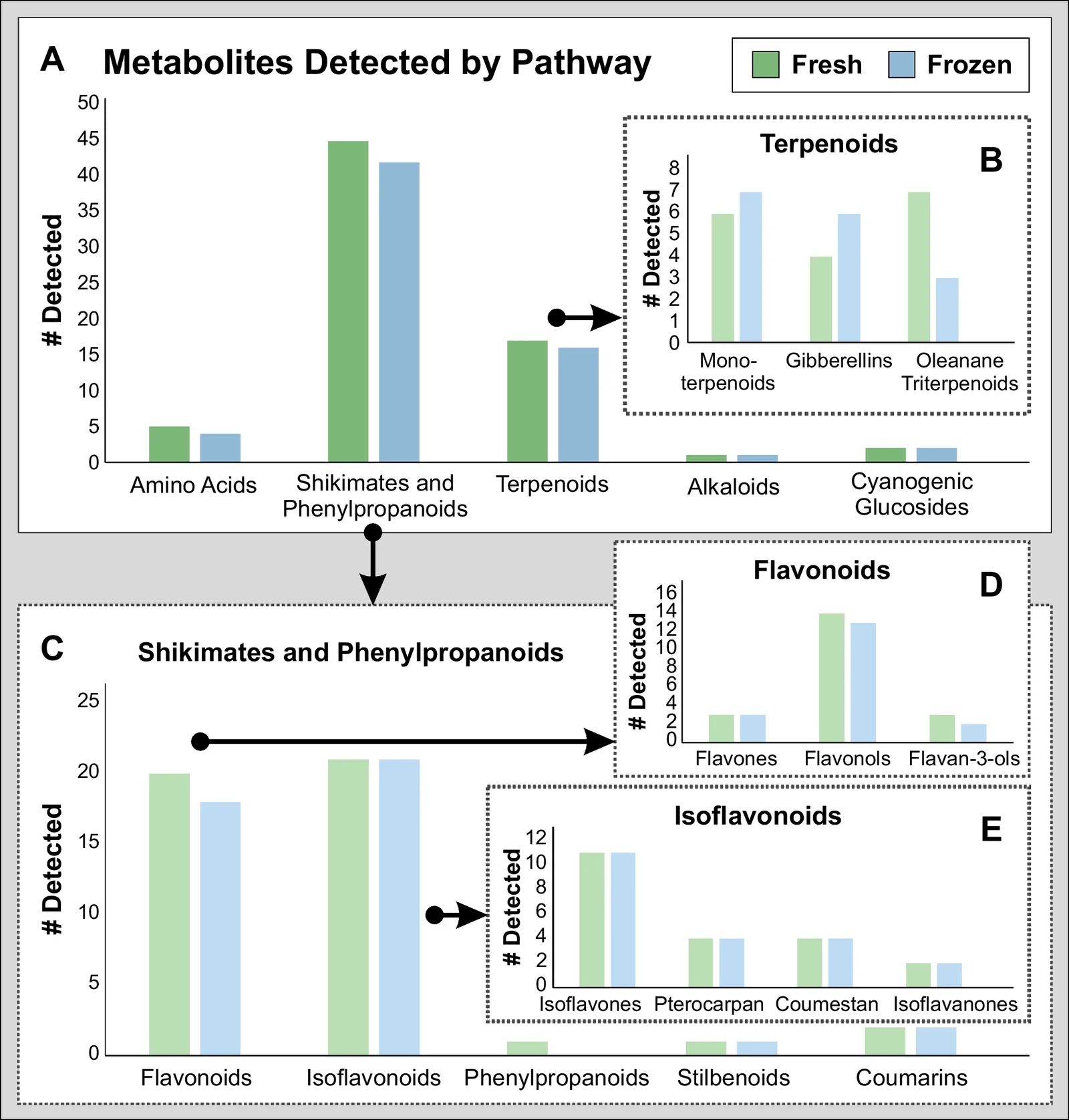
Number of *T. repens* metabolites detected by fresh (green) and frozen (blue) analysis. **A** Analytes are classified by major pathways, and highly detected categories including **B** terpenoids and **C** shikimates and phenylpropanoids are further subdivided. The two largest classes of shikimates and phenylpropanoids, **D** flavonoids and **E** isoflavonoids, are further subdivided. Metabolites were compiled from the KNApSAcK database using the search “*Trifolium repens*” and were considered detected if at least one adduct ([M + H]^+^, [M + Na]^+^, and/or [M + K]^+^) was detected with little-to-no off-tissue signal for at least one sample. Images with spotty spatial localization were omitted

Frozen analysis had minimal impact on the classes of metabolites detected. In Fig. 4A, which investigates the known *T. repens* metabolites detected by each method in relation to metabolic pathways, both methods detected the same number of alkaloids and cyanogenic glycosides, but fresh tissue analysis detected a slightly higher total number of terpenoids, shikimates/phenylpropanoids, and amino acids. Detection of terpenoids appeared most impacted by the method of analysis, and the class of terpenoid further impacted detection; in Fig. 4B, frozen analysis detected slightly more monoterpenoids and gibberellins, while fresh analysis detected more oleanane triterpenoids. Within the shikimate and phenylpropanoid pathway in Fig. 4C, the only superclasses with differences in detection were flavonoids and phenylpropanoids. Phenylpropanoids were only detected in fresh analysis, and fresh analysis also detected slightly more flavonoids, particularly flavanols and flavan-3-ols (Fig. 4D). Isoflavonoids were the most consistently detected superclass, and detection was consistent between methods and across all classes (Fig. 4E). Overall, frozen analysis appears to be beneficial for detecting monoterpenoids and gibberellins but less effective for detecting oleanane triterpenoids. This result warrants further investigation into differences in the character of ablation plumes between fresh and frozen analysis.

**Fig. 5:**
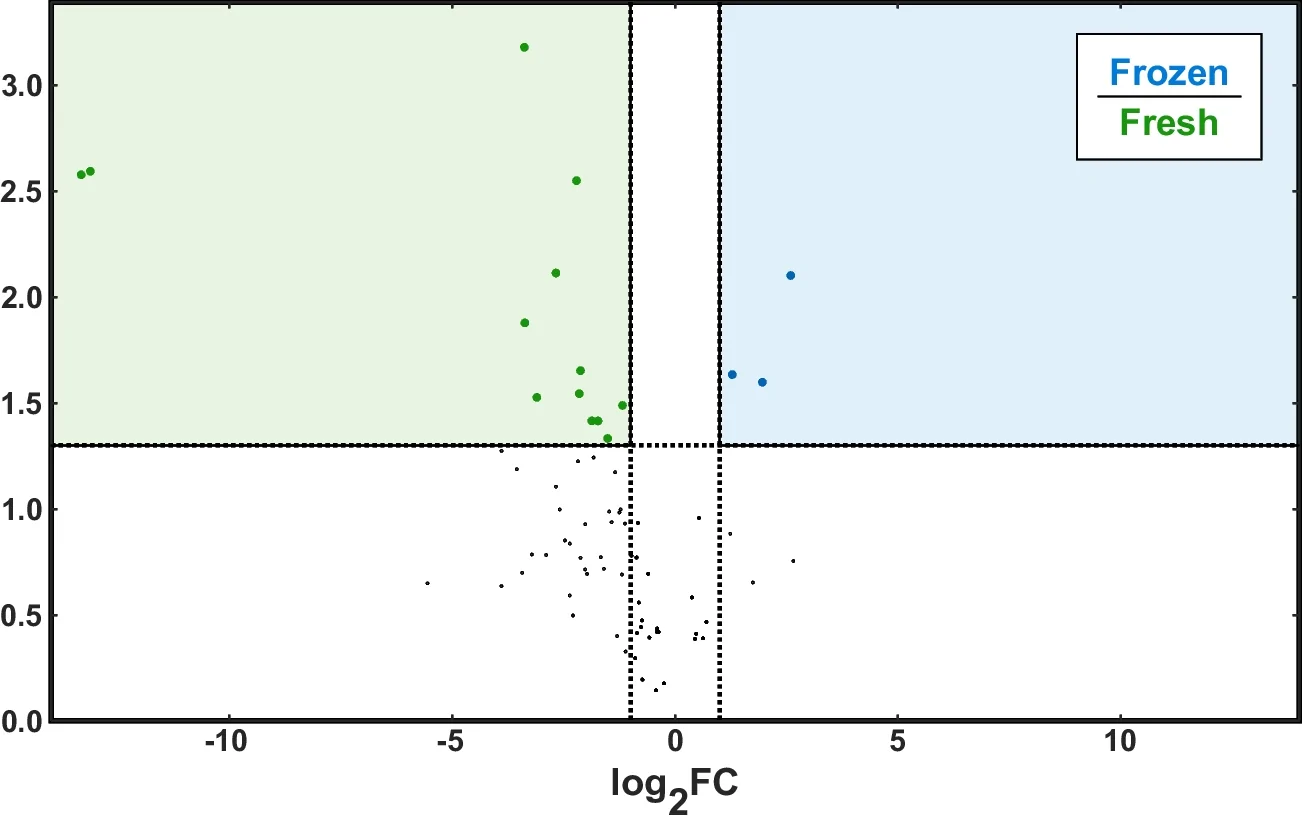
Volcano plot of the abundance of *T. repens* metabolites normalized to average volume ablated for frozen (blue) vs. fresh (green) methods. Features include the [M + H]^+^, [M + Na]^+^, and [M + K]^+^ adducts of metabolites from the KNApSAcK database search for “*Trifolium repens*,” but ion images were generated and screened for each feature to remove those with high off-tissue abundance (particularly background from tape and/or CMC), spotty spatial localization, and/or low abundance across all samples. In this conservative estimate, 71/198 features remain. Data and tentative identifications for significant features are provided in Table SI. For practical applications, the volcano plot and data table for raw abundance values are also provided in Fig. S3 and Table SII, respectively

Although the total number of metabolites detected was similar across all classes, the frozen analysis was overall slightly less sensitive than the fresh analysis as shown in the volcano plot in Fig. 5. This plot evaluates ion abundance normalized to the average calculated volume of ablation spots for each method. Of the metabolites demonstrating a significant fold change in abundance between methods, 13/16 were less abundant in the frozen analysis. While frozen IR-MALDESI analysis is known to enhance detection of metabolites in cryosectioned mammalian tissue [47], the increased abundance is a product of the external ice matrix, which is avoided in this method due to potential matrix effects from inconsistent deposition across the cuticle. As such, the decrease in sensitivity for frozen leaf tissue is likely an artifact of the decreased diameter of the ablation spots. The inverse relationship between ion abundance and spatial resolution is well-documented in MSI analysis—increasing spatial resolution by decreasing the diameter of ablation spots results in decreased abundance due to the smaller amount of material sampled from each spot [41, 47 ]. As with any MSI method, advantages and limitations must be weighed for individual experiments; in this case, frozen tissue analysis enables increased spatial resolution but slightly decreases overall ion abundance.

**Fig. 6:**
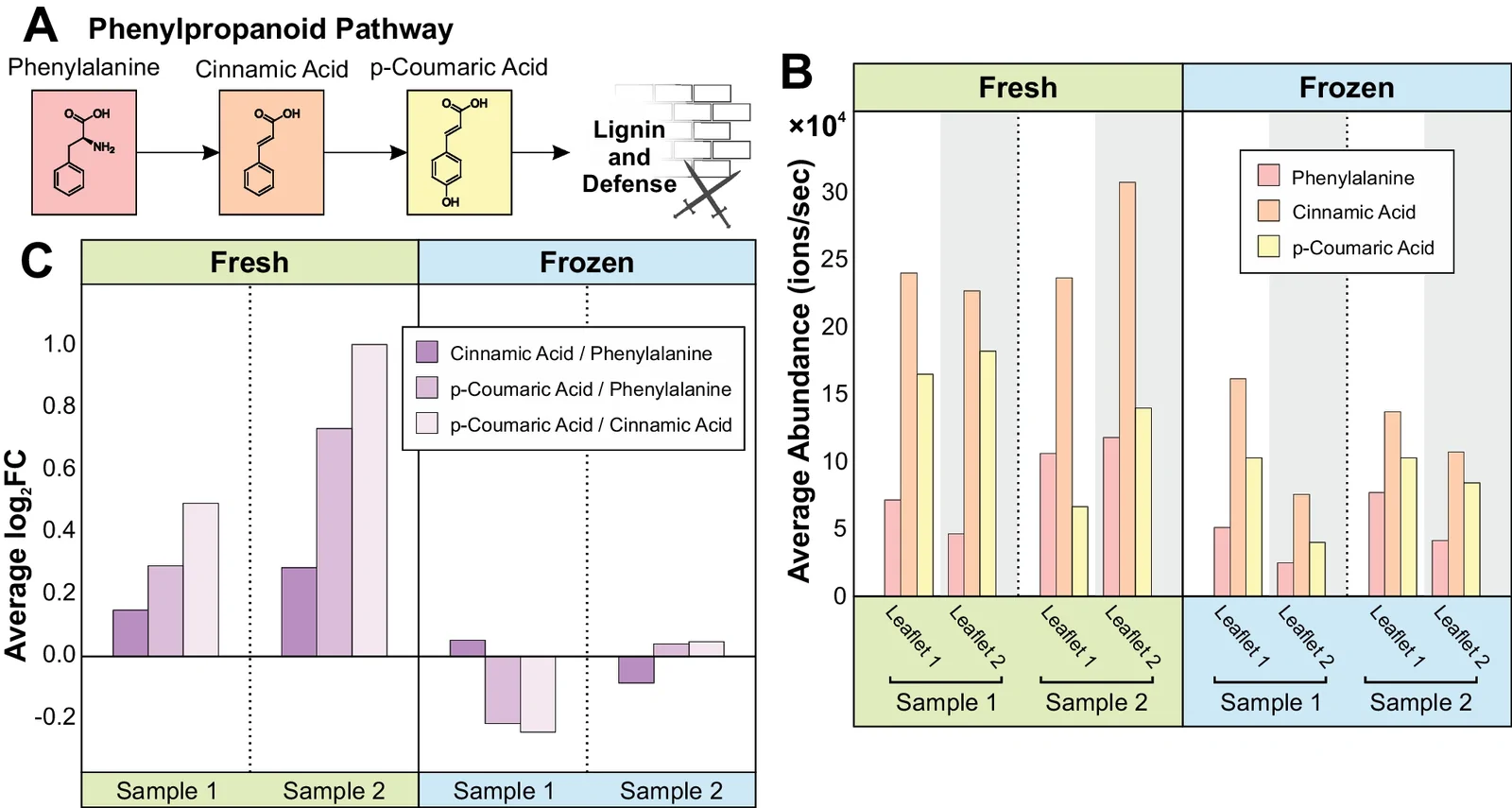
**A** In an early response to wounding, plants activate the phenylpropanoid pathway to produce lignin and/or secondary metabolites for defense. This pathway begins by converting phenylalanine to cinnamic acid and then converting cinnamic acid to p-coumaric acid. **B** The average abundances of the protonated adducts of phenylalanine (*m*/*z* 166.0863), cinnamic acid (*m*/*z* 149.0597), and p-coumaric acid (*m*/*z* 165.0546) in leaflet one (analyzed first) and leaflet two (analyzed second) of each sample were plotted for fresh (left, green) and frozen (right, blue) analyses. **C** For fresh (left, green) and frozen (right, blue) analyses, log_2_FC values were plotted for the change in average normalized abundance between leaflets one and two of each sample. Normalization was conducted voxel-by-voxel for (1) cinnamic acid normalized to phenylalanine (dark), (2) p-coumaric acid normalized to phenylalanine (medium), and (3) p-coumaric acid normalized to cinnamic acid (light). This normalization highlights areas of increased metabolism and/or storage of early phenylpropanoid metabolites

The trend toward decreased ion abundance for frozen analysis could be explained by the decreased ablation diameter; however, it may also be influenced by the intentional quenching of metabolic processes. Interestingly, most features that demonstrated a significant reduction in ion abundance were sodiated (7/13), and one feature, tentatively identified as soyasapogenol C, was significantly more abundant in the fresh method in its sodiated form but significantly more abundant in the frozen method in its protonated form. This discrepancy suggests that results may be influenced by inconsistent salt concentrations; thus, metabolites with a significant fold change in the same direction for at least two adducts were considered with greater confidence. Only one feature, tentatively identified as kaempferol 3-O-galactoside, met this criterion. Kaempferol 3-O-galactoside has a documented relationship to stress response in plants [48], as do many of the other tentatively identified features [49–53]. For many stress-induced defense metabolites, significant accumulation is only documented to occur on a timescale far exceeding that of these experiments, but most time studies analyze bulk extractions and study a limited number of metabolites and species using a limited number of time points. As such, it is likely that the trend toward decreased ion abundance results from a combination of both quenched metabolic processes and decreased ablation diameter, but it is difficult to ascribe this trend to a single cause. To more directly explore metabolic differences resulting from stress, the effects on several well-studied metabolites crucial to early stress responses were evaluated in Fig. 6 by comparing ion abundances in leaflets analyzed chronologically. For each sample, differences in ion abundances between the leaflets analyzed first and second could indicate a stress response; each leaflet that was analyzed second had approximately 30 min to respond to stress while the first leaflet was being analyzed.

Mechanical wounding of leaves initiates stress signaling responses via reactive oxygen species (ROS) and activation of the jasmonate (JA) signaling pathway [54]. While neither of these responses could be measured directly (ROS are outside of the *m*/*z* range and JA signaling metabolites overlapped with background isomers/isobars), most metabolomics studies focus on specialized secondary metabolites such as flavonoids. Flavonoids are synthesized through the phenylpropanoid pathway, and JA signaling is known to upregulate genes involved in this pathway, sometimes in a matter of minutes [32, 55 ]. Since activation of the phenylpropanoid pathway results from mechanical wounding and affects metabolites commonly evaluated in plant metabolomics studies, effects on this pathway were considered most relevant. The phenylpropanoid pathway begins with the deamination of phenylalanine to cinnamic acid and subsequent hydroxylation of cinnamic acid to p-coumaric acid; the plant then uses these precursors to synthesize monolignols and/or specialized secondary metabolites for defense [30] as summarized in Fig. 6A. In Fig. 6B, the average abundances of phenylalanine, cinnamic acid, and p-coumaric acid were calculated for each leaflet of each sample analyzed by each method; representative spectra are available in Fig. S4. Differences between leaflet one and leaflet two were not consistent across the two samples analyzed by the fresh method, but the average abundance consistently decreased for all three metabolites in both samples analyzed by the frozen method. Freezing quenches metabolic processes, so this is most likely an artifact of the method itself and may be related to scattered deposition of ice crystals over the course of analysis; in IR-MALDESI analysis of mammalian tissue with the reflective objective, frozen and thawed tissue had comparable ablation diameters, but frozen tissue with an ice matrix had a smaller ablation diameter [41]. As such, scattered ice crystals building up over the course of analysis may have led to decreased ablation diameter in the affected areas, resulting in decreased ion abundance.

To more directly evaluate the conversion of phenylalanine to cinnamic acid and p-coumaric acid, the abundances of cinnamic acid and p-coumaric acid were normalized to the abundance of phenylalanine at each location. Changes in the average normalized abundances between leaflets are summarized for all samples in a fold change plot in Fig. 6C. The log_2_FC was positive for both fresh samples for both cinnamic acid (0.15, 0.29) and p-coumaric acid (0.49, 1.00), while the log_2_FC was inconsistent between frozen samples for both cinnamic acid (0.05, − 0.09) and p-coumaric acid (− 0.24, 0.05), suggesting that more phenylalanine was converted to cinnamic acid and p-coumaric acid between leaflets one and two in fresh analysis. This difference suggests that the frozen method prevented early metabolic changes from stress over the course of MSI analysis.

A final concern for the new method is delocalization, wherein metabolites migrate during sample preparation, reducing the biological accuracy of the resulting ion images. Freeze–thaw cycles are a main concern: freezing causes cells to burst, and subsequent thawing allows the cell contents to migrate [14]. Leaf tissue is water-rich, so freezing and thawing can result in significant delocalization. In embedded samples, hydrophilic metabolites can also delocalize by diffusing into the embedding medium [56], although the hydrophobic barrier of the leaf cuticle may offer some protection compared to other sample types. A 2018 study by Li et al. found 10% gelatin to be the optimal embedding medium for reducing delocalization in ginkgo leaf cross-sections [14]; however, gelatin must be heated to maintain its viscosity. Since heating would be counterproductive for the purposes of this protocol, CMC was selected to mount the sample since it was the second-best option for preventing delocalization in Li et al., 2018. No thawing was visible during analysis, and the samples appeared to stay structurally intact as seen in Fig. 1, so potential sources of delocalization in this protocol should be limited to diffusion into the CMC gel used to mount the sample and migration of metabolites during ice crystal formation, particularly in the vascular tissue.

**Fig. 7:**
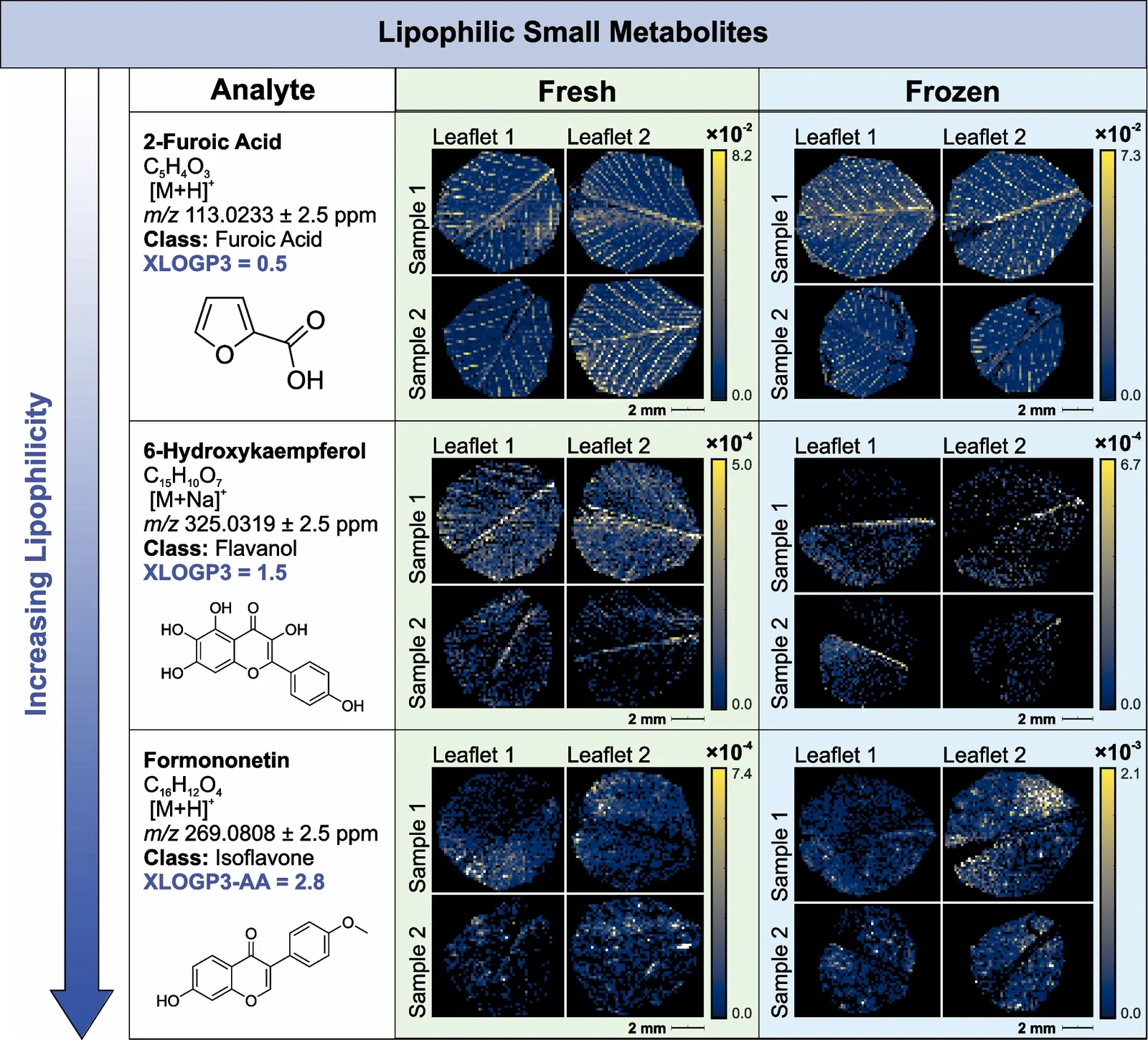
TIC-normalized ion images of three lipophilic small metabolites (left column) from fresh (middle column) and frozen (right column) MSI analyses. The analytes are listed in order of increasing predicted logP from top to bottom. The name, molecular formula, adduct, *m*/*z*, class, predicted logP, and structure are provided in the left column. In each set of four ion images: the first sample is on the top row, and the second sample is on the bottom row; the leaflet analyzed first is in the left column, and the leaflet analyzed second is in the right column

**Fig. 8:**
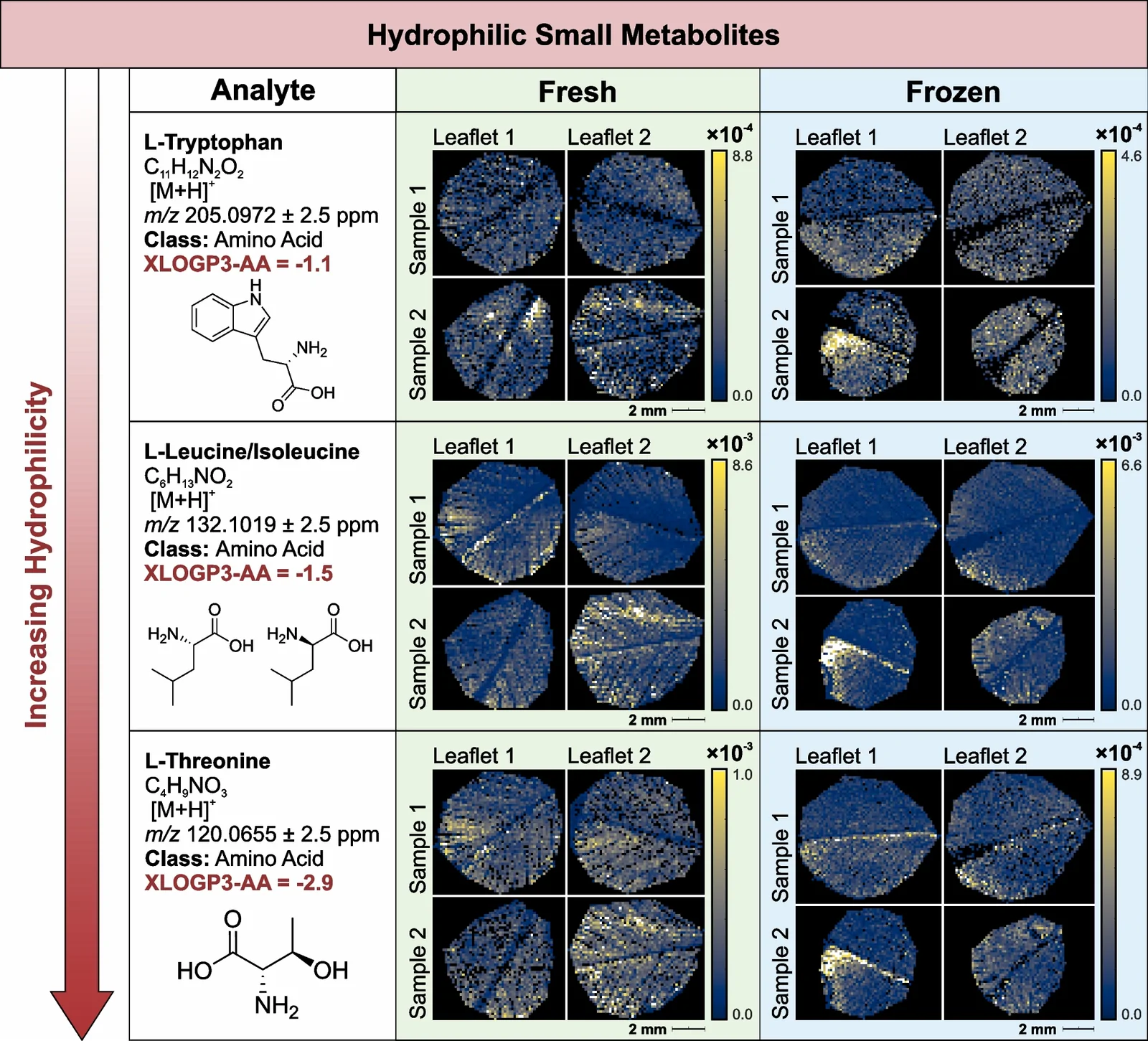
TIC-normalized ion images of three hydrophilic small metabolites (left column) from fresh (middle column) and frozen (right column) MSI analyses. The analytes are listed in order of decreasing logP from top to bottom. The name, molecular formula, adduct, *m*/*z*, class, predicted logP, and structure are provided in the left column. In each set of four ion images: the first sample is on the top row, and the second sample is on the bottom row; the leaflet analyzed first is in the left column, and the leaflet analyzed second is in the right column

To check for potential delocalization, ion images were generated for three representative lipophilic small metabolites in Fig. 7 and three representative hydrophilic small metabolites in Fig. 8. The images were TIC-normalized to highlight differences in localization patterns rather than abundance. For each metabolite, patterns of spatial distribution were compared between methods. The fresh method provides a baseline for the localization of metabolites, since the sample was never frozen and was mounted on tape rather than CMC. The patterns of spatial localization were conserved between the fresh and frozen methods and align with current literature, indicating that the structural integrity of the sample and localization of metabolites were maintained.

In Fig. 7, 2-furoic acid has a low positive predicted logP (XLOGP3 = 0.5), making it the least lipophilic of the lipophilic metabolites evaluated. It appeared to be distributed throughout the tissue and was most abundant in the veins. This pattern was conserved across both methods and aligns with literature indicating that it is localized to vascular tissue [57] and related to water tolerance [58]. Vascular tissue has a higher water content than the surrounding lamina, so consistently resolving this pattern is particularly promising, especially since 2-furoic acid is the least lipophilic metabolite—the relative hydrophilicity and localization to tissue with high water content suggest it would be the most likely to suffer from delocalization due to freeze–thaw cycles or ice crystal formation. A more lipophilic metabolite, 6-hydroxykaempferol (XLOGP3 = 1.5), was also most abundant in vascular tissue, but was much more abundant in primary veins. The smaller veins were harder to differentiate in the frozen method; however, the high abundance in primary veins suggests that this is an artifact of inconsistent detection from the overall lower abundance in frozen analysis. In both methods, the most lipophilic of the metabolites, formononetin (XLOGP3-AA = 2.8), was distributed across most of the tissue but is highly abundant in scattered areas. This pattern makes sense in the context of formononetin accumulation as a long-term stress response in areas wounded by insects and pathogens [59]. The consistency of these spatial localization patterns across methods supports that frozen analysis does not cause delocalization of lipophilic metabolites.

Patterns of spatial localization for increasingly hydrophilic amino acids were also similar between the fresh and frozen methods. In Fig. 8, the least hydrophilic amino acid, L-tryptophan (XLOGP3-AA =  − 1.1), was widely distributed across the lamina and was also highly abundant in certain areas. Like many amino acids, L-tryptophan is broadly involved in plant metabolism, but it is also important for crosstalk between stress hormones and biosynthesis of metabolites that defend against stress [60]; therefore, this pattern of distribution is expected, and the areas of high abundance likely correlate to long-term stress responses. This spatial pattern was consistent between methods. The two more hydrophilic amino acids L-leucine/L-isoleucine (XLOGP3-AA =  − 1.5) and L-threonine (XLOGP3-AA =  − 2.9), were also widely distributed across the lamina but showed even lower relative abundance in the vascular tissue. These amino acids are similarly broadly involved in metabolism and known to accumulate in response to stress [61], so this spatial distribution pattern aligns with literature and was also conserved between methods, although L-threonine was less abundant overall, resulting in less consistent detection. The similar patterns of spatial distribution of these amino acids between the fresh and frozen methods further support that even hydrophilic small molecules experience minimal delocalization.

## Conclusion

An effective method was established for IR-MALDESI MSI of leaf tissue wherein the sample was frozen in-source immediately after harvest and remained frozen at 1–2% humidity throughout analysis. This method reduced ablation spot diameter to ~ 44 µm, enabling increased spatial resolution with a conventional 2.97-µm IR laser. Detection remained comparable across most metabolites with a slight decrease in abundance resulting from smaller ablation spots. No evidence of delocalization was found, including for amino acids, which are particularly susceptible as hydrophilic small metabolites. Although differences in ablation spot diameter complicated the evaluation of stress responses between fresh and frozen analysis methods, literature maintains that freezing the sample as soon as possible after harvest reduces unwanted stress responses, and changes in the abundance of several metabolites from the beginning of the phenylpropanoid pathway suggest that post-harvest metabolic changes were reduced by the frozen method. These combined benefits position IR-MALDESI MSI of frozen leaf tissue as an ideal method when spatial resolution must be increased but UV-MALDESI is not preferable (e.g., when flavonoids are inconsistently distributed or complete ablation is required) or when it is imperative to minimize stress responses to preserve the metabolic state of the sample at the time of harvest.

## Supplementary Information

Below is the link to the electronic supplementary material.

Supplementary file1 (DOCX 4.87 MB)

## Data Availability

Raw datafiles (.RAW, .mzML, and .imzML files) for all samples and a.xlsx compilation of KNApSAcK annotation data, additional relevant information, and representative MS1 spectra for all detected metabolites are freely available through Dryad at https://doi.org/10.5061/dryad.p8cz8wb6r.
