# Supplementary material for: In-source freezing of Trifolium repens leaf tissue to increase spatial resolution and quench stress responses during IR-MALDESI mass spectrometry imaging
Source: Anal Bioanal Chem. 2026 Aug 3;418(21):7139–52. doi: 10.1007/s00216-026-06710-z (PMC13658390; doi:10.1007/s00216-026-06710-z)
Supplement: Supplementary file 1 [file 216_2026_6710_MOESM1_ESM.docx]

**SUPPLEMENTAL MATERIALS**

**In-Source Freezing of *Trifolium repens* Leaf Tissue to Increase Spatial Resolution and Quench Stress Responses During IR-MALDESI Mass Spectrometry Imaging**

Quinn Mills and David C. Muddiman^*^

*Biomolecular Imaging Laboratory for Disease and Exposure Research (BILDER), Department of Chemistry, North Carolina State University, Raleigh, NC*

**Submitted to:** *Analytical and Bioanalytical Chemistry*

**Article Type:** Research Article

**Submitted:** May 22, 2026

**Revised I:** July 21, 2026

**Manuscript:** 29 Pages / 8 Figures

**Supplemental Material:** 5 Pages / 4 Figures / 2 Tables / 1 Spreadsheet

**Keywords:** IR-MALDESI, Mass Spectrometry Imaging, Plant Imaging, Plant Metabolomics, Plant Stress Response

***Author for Correspondence**

David C. Muddiman, Ph.D.

Department of Chemistry

North Carolina State University

Email: [dcmuddim@ncsu.edu](mailto:dcmuddim@ncsu.edu)

**Figure S1.** Preliminary data from fresh and frozen analysis of collard leaves. Images show ablation spots at 5X magnification for **(A)** fresh and **(B)** frozen analysis, and 10X magnification for **(C)** fresh and **(D)** frozen analysis. Data were collected using the same protocol and conditions described in this study. Although these preliminary results were more significant, ablation appeared incomplete for frozen analysis, likely due to the thickness (~1 mm) of the leaves. Clovers were chosen for the final study since their thin (~0.1 mm) leaves reduce the possibility of ablation spots appearing smaller due to incomplete ablation rather than an actual reduction in ablation spot diameter

**Figure S2.** A leaflet after fresh analysis mounted on CMC. After analysis, it was noted that the leaf and CMC had both dried out, shrinking the tissue away from the height accounted for by the AzC and therefore pulling the sample surface out of laser focus. The ablation spots in the image got progressively less visible since the tissue shrank farther out of focus over time. While the resulting ion images could not be directly compared to those from the fresh and frozen methods, spectra from the top left portion of the ROI were used to evaluate possible ion suppression from the CMC mounting medium

**Table SI.** Description of significant volcano plot features for volume-normalized abundance. Green rows denote features more abundant in fresh analysis, and blue rows denote features more abundant in frozen analysis. Protonated adducts are bolded, and features with multiple adducts are italicized.

**Figure S3.** Volcano plot of the raw abundance of *T. repens* metabolites for frozen (blue) vs. fresh (green) methods. Features include the [M+H]^+^, [M+Na]^+^, and [M+K]^+^ adducts of metabolites from the KNApSAcK database search for “*Trifolium repens*,” but ion images were generated and screened for each feature to remove those with high off-tissue abundance (particularly background from tape and/or CMC), spotty spatial localization, and/or low abundance across all samples. In this conservative estimate, 71/198 features remain. Since the abundance values in this plot were not normalized to ablation volume, it does not account for the decreased volume ablated in the frozen method but is more applicable for evaluating practical effects of applying the frozen method.

**Table SII.** Description of significant volcano plot features for raw abundance data. Green rows denote features more abundant in fresh analysis, and blue rows denote features more abundant in frozen analysis. Protonated adducts are bolded, and features with multiple adducts are italicized.

**Figure S4.** Reflective plots of representative mass spectra highlighting the [M+H]^+^ adducts of the biosynthetic precursors of lignin from **Figure 6** in **(A)** leaflet one of fresh sample 2, **(B)** leaflet 2 of fresh sample 2, **(C)**leaflet one of frozen sample 2, and **(D)** leaflet 2 of fresh sample 2. The calculated *m/z* values are as follows: phenylalanine = *m/z* 166.0863, cinnamic acid = *m/z* 149.0597, and p-coumaric acid = *m/z* 165.0546.
